# Factor structure and predictors of causal beliefs about seven mental illnesses among the Singapore general population

**DOI:** 10.3389/fpubh.2025.1612820

**Published:** 2025-07-09

**Authors:** Yoke Boon Tan, Eng Hong Tay, Shazana Shahwan, Savita Gunasekaran, Benedict Wei Zhi Lim, Bernard Chin Wee Tan, Wei Jie Ong, Saleha Shafie, Edimansyah Abdin, Porsche Poh, Georg Schomerus, Siow Ann Chong, Mythily Subramaniam

**Affiliations:** ^1^Research Division, Institute of Mental Health, Singapore, Singapore; ^2^Silver Ribbon, Singapore, Singapore; ^3^Department of Psychiatry and Psychotherapy, University of Leipzig Medical Center, Leipzig, Germany; ^4^Saw Swee Hock School of Public Health, National University of Singapore, Singapore, Singapore

**Keywords:** causal beliefs, factor structure, general population, mental health literacy, mental illness

## Abstract

**Introduction:**

Knowledge about the causes of mental illness is a crucial component of mental health literacy. Poor understanding of the etiology of mental illness can lead to stigmatizing behaviors and hinder access to appropriate help. The current cross-sectional study examined the general population’s causal beliefs about seven mental illnesses and explored the factor structure of the revised causal beliefs scale.

**Methods:**

Four thousand one hundred ninety-five respondents were randomly assigned a vignette and were asked questions on their recognition of the mental illness depicted, causal beliefs, and prior experience related to the mental illness (similar problems, had a mental health-related job or family and friends with similar problems). Exploratory and confirmatory factor analyses were used to examine the factor structure of the causal beliefs scale. Multivariable linear regression was used to examine its predictors.

**Results:**

Factor analyses revealed a four-factor structure of causal beliefs: physical, psychosocial, personality, and biogenetic. Causal beliefs differed across the vignettes. Compared to schizophrenia, biogenetic beliefs were less likely to be endorsed for all mental illnesses except dementia, while personality beliefs were more likely to be endorsed for depression with suicidality. Compared to respondents who did not recognize the mental illness, those with correct recognition were more likely to endorse biogenetic and psychosocial beliefs, and less likely to endorse physical and personality beliefs.

**Discussion:**

Factor structure of the original causal beliefs scale was found to be valid in the Singapore population. Individuals who correctly recognized the mental illness appeared to comprehend its etiology well. However, some misconceptions about the etiology of mental illness persist, particularly with regards to relating obsessive-compulsive disorder to physical factors (e.g., virus) and depression with suicidality to personality flaws. As future awareness campaigns continue to address the gaps in literacy levels, careful promotion of certain causal beliefs is crucial to avoid conveying unintended messages.

## 1 Introduction

Personal beliefs about the causes of mental illness are an important component of mental health literacy (MHL) (1). These beliefs shape individuals’ attitudes and behaviors toward people with mental illness and help-seeking beliefs (2–4). As such, the lack of awareness or poor understanding of the etiology of mental illness can result in undesirable outcomes such as public stigma and social distancing (5, 6) and become a barrier to accessing appropriate help (7).

Research on causal beliefs have explored many forms of explanations for mental illness and a number of measures have been developed to evaluate them (8). In contrast to professionals working in the mental health field, general populations tend to endorse psychosocial causes over biogenetic causes, though biological explanations are increasingly endorsed by the public as shown in more recent studies (4), especially for schizophrenia (9–11). Other causal beliefs include personal attributes and supernatural causes (7, 11, 12). According to sociocultural research, different cultures can share similar beliefs on the etiology of mental illness. For instance, a systematic review of 36 studies found that stress due to socio-environmental factors was consistently perceived as the top cause for depression across Western and non-Western cultures (13). In contrast, beliefs originating from traditional and supernatural ideas vary across cultures and are more commonly found in Asian and African cultures (13, 14), particularly for psychotic disorders (15). These beliefs are seemingly independent of the country of residence, as seen from ethnic groups of South Asian descent in the United Kingdom having more beliefs in supernatural causes for psychosis than the White British (16).

Social contact theory suggests that causal beliefs are formed and influenced by experiences and personal contact with people with mental health conditions (17). For instance, Pullen et al. (18) studied the effects of social network and found that individuals who knew family members or friends with mental health problems were more likely to recognize the symptoms experienced by the vignette character as a mental illness. In the study, the likelihood of attributing mental illness to biogenetic causes and less to poor character increased with the number of known family members and friends with mental health problems. Other studies mostly focused on schizophrenia and found that views on the etiology of the illness can vary among groups of different backgrounds and contact history (15, 19). In these studies, patients generally endorsed a mix of biological and psychological causes, whereas relatives endorsed psychosocial causes only. Similar to patients, mental health professionals endorsed both types of causes but showed stronger inclination toward biological causes, due to their academic training and experiences gained from direct contact with patients (20).

Singapore is a multi-ethnic city state in Southeast Asia with a resident population of 4.18 million, comprising 74.0% Chinese, 13.5% Malay, 9.0% Indian, and 3.4% other ethnic groups (21). In 2015, a nationwide study was conducted to establish MHL levels among the general population. Five common mental disorders in Singapore – depression, schizophrenia, obsessive-compulsive disorder (OCD), alcohol abuse and dementia – were selected for investigation. These mental health conditions were depicted in the form of vignettes and the overall correct recognition of the conditions was 43.7% (22). A three-factor structure underlying the population’s beliefs about the causes of mental illness was found, with 97.7% of respondents endorsing psychosocial causes, 83.5% endorsing personality causes, and 37.0% endorsing physical causes (23).

The present study is the second nationwide study that reviews the current MHL landscape in Singapore. To enable tracking and comparisons of MHL in the general population, the five mental health conditions in the 2015 study were retained. Prevalence data from the latest population-level epidemiological surveys in Singapore (i.e., Singapore Mental Health Study 2016 and Well-being of the Singapore Elderly 2023) revealed that these conditions remained as the most common mental disorders in Singapore, with lifetime prevalence of 6.3% for depression, 2.3% for schizophrenia (and other psychotic disorders), 3.6% for OCD, 4.1% for alcohol abuse and 8.8% for dementia (24–26). Two other mental health conditions – suicidality and gambling disorder – that were added as vignettes in the current study showed lifetime prevalence of 7.8% and 2.7%, respectively (27, 28). The high treatment gap in the general population (78.6%) is also a growing concern for public health (29), with lack of knowledge commonly cited as a contributing factor. Between the first and the current study, a number of mental health campaigns and initiatives were implemented to raise the public’s awareness on the symptoms and etiology of mental illness. Most notably, the second phase of the nationwide movement ‘Beyond the Label’ by the National Council of Social Services launched in 2022 continues to improve public mental health knowledge and address mental health stigma through fostering partnerships with agencies and organizations as well as greater community engagement such as public education campaigns, peer support skills training, and mental wellness workshops for companies and schools. Mindline.sg, Singapore’s national digital platform for mental health and a first-stop touchpoint for the public, was also initiated in 2020 with features including a service wayfinding tool, self-help resources, online forums and chatbot that provide information, support and access to professional help. With these ongoing efforts to raise awareness, public beliefs about the causes of mental illness are expected to improve, with endorsement of psychosocial causes to remain high but a reduction in physical and personality beliefs in particular. Evaluating MHL on a population level periodically is therefore crucial to assess whether public knowledge about mental illness have improved and to identify gaps to address misinformation and misconception, and improve understanding. This study aimed to: (i) report the public’s causal beliefs of seven mental illnesses, (ii) review the factor structure of the revised causal beliefs scale, and (iii) examine the correlates of causal beliefs in the Singapore general population.

## 2 Methods

### 2.1 Participants and procedures

Data were taken from the Mind Matters 2022–2023 study conducted between September 2022 and February 2024 in Singapore. This second nationwide study on MHL replicates the methodology of first study in 2015 (22). The study population included Singapore Citizens and Permanent Residents aged 18–65 years living in Singapore at the time of the survey. A disproportionate stratified sampling design was used for the study, with 12 strata defined by age group (18–34, 35–49, and 50–65) and ethnicity (Chinese, Malay, Indian, and Other ethnic groups). 50–65 age group, Malay and Indian ethnicity were oversampled to ensure adequate sample size for subgroup analysis. The sample was derived using the sample frame from a national administrative database in Singapore that maintains data on age, gender, ethnicity and residential addresses of all residents. Sample size calculations were based on the overall prevalence of correct recognition of mental disorders in Singapore as determined in the first study (22). Factoring in an estimate of 10% missing data, a sample size of 590 respondents per vignette was estimated, resulting in a total target sample size of 4,130 respondents for the study.

Face-to-face interviews were administered by trained lay interviewers in English, Chinese, Malay or Tamil. Respondents were given monetary compensation of SGD$40 as a token of appreciation for their time and effort. Written informed consent was obtained from all respondents and from parents/guardians of respondents aged 18–20 years. Ethics approval was obtained from the institutional ethics committee, the National Healthcare Group Domain Specific Review Board (No. 2021/00695).

### 2.2 Measures

The study used a vignette approach whereby respondents were randomly assigned and presented one out of seven vignettes depicting a fictional character with a specific mental illness. The name of the character (denoted as XX in this paper) was matched to the respondent’s gender and ethnicity to facilitate identification with the character (e.g., “Mr Tan” was shown to Chinese male respondents, “Siti” for Malay female respondents, and “Devi” for Indian female respondents). Vignettes used in this study included the five vignettes (on depression, schizophrenia, OCD, alcohol abuse and dementia) from the previous study (22) and two newly developed vignettes (one on depression with suicidality and the other on gambling disorder).

#### 2.2.1 Recognition of mental illness in vignette

Respondents were asked an open-ended question on what they thought the character was suffering from and were probed to name the condition causing the symptoms where necessary. Responses were coded as “correct recognition” if respondents were able to correctly label the specific condition, “mislabeled/unspecified mental illness” if respondents answered other mental illness or gave a general response of mental illness/disorder, or “did not recognize” if respondents mentioned responses such as stress, insomnia and sadness or did not think the symptoms were a problem to the character. The coding process followed the same structure from the first study where each vignette is coded by two team members independently and discrepancies were subsequently discussed as a team with the senior researchers until a consensus was reached (22).

#### 2.2.2 Causal beliefs about mental illness

Respondents were asked about the likelihood of 12 items being the cause of the problem described in the vignette on a 5-point Likert scale (very likely, likely, depends, unlikely, and very unlikely). The first study included nine out of the 10 items from the causal beliefs scale developed by Reavley and Jorm (11) and one other item on supernatural causes (‘How likely is it that these sorts of problems are caused by spirit possession, supernatural causes or black magic?’). The current study included the full 10 items from the causal beliefs scale, the one item on supernatural causes, and one new item (‘How likely is it that these sorts of problems are a punishment, retribution or karma for some previous wrongdoing or bad deeds by the person or the person’s family?’).

#### 2.2.3 Prior experience with mental illness

Individuals’ experiences with the mental illness illustrated in the vignette were assessed using three yes/no questions: “Have you ever had problems similar to XX’s?,” “Have you ever had a job that involved providing treatment or mental health services to a person with a problem like XX?,” and “Has anyone in your family or close circle of friends ever had problems similar to XX’s?”.

#### 2.2.4 Sociodemographic variables

Sociodemographic information about age, gender, ethnicity, marital status, highest education level, employment status, and monthly income were collected.

### 2.3 Data analysis

All estimates were weighted to adjust for oversampling and post-stratified for age and ethnicity distributions between the survey sample and the Singapore resident population in the year 2024. Sociodemographic data and linear regression were performed on STATA S/E Version 15.0 (30), while factor analyses were performed on Mplus (Version 8.0) (31). Sample weights were included in all analyses.

As new items were added to the set of causal beliefs questions, exploratory factor analysis (EFA) and confirmatory factor analysis (CFA) to analyze the factor structure of the revised scale were conducted. The sample was first randomly split into two equal proportions. In line with the original authors of the casual beliefs scale (11), maximum likelihood with robust standard errors (MLR) was used for the estimator and geomin oblique rotation was used to allow for correlation between factors. Missing data were handled using full information maximum likelihood (FIML). EFA was performed on one sample to identify the number of factors and factor loadings. The following criteria were used to determine the number of factors to be included: visualization and inspection of scree plot, eigenvalues>1, and factor loadings>0.30 (32). Thereafter, CFA was performed on the remaining sample to confirm the factor structure identified in the EFA. Criteria for the model fit indices include: RMSEA < 0.05, TLI/CFI > 0.90, SRMR < 0.05, and *χ*^2^ statistic (33). Cronbach’s alpha was calculated to measure the internal consistency.

Responses to each item on the causal beliefs scale were first dichotomized to distinguish respondents who endorse the item (1 = very likely or likely) and respondents who do not endorse the item (0 = depends, unlikely, or very unlikely). Missing values were addressed using listwise deletion method. A total score for each factor as determined by the factor analyses was then created by summing the scores of the relevant items. These total scores were subsequently used in the computation of weighted mean and standard deviation (SD) and in the regression analyses. Proportion of causal beliefs endorsed by vignette (n and weighted percentage) were generated separately. Multivariable linear regression analyses were conducted on the overall sample to examine the associations between the dependent variables which include the factors of causal beliefs (as established from the factor analyses) with the independent variables which include sociodemographic variables, type of vignette, recognition of mental illness and prior experience with mental illness. Variance inflation factor (VIF) was also performed, where VIF values for all independent variables were below 5, suggesting little to no multicollinearity between the variables.

## 3 Results

Eight thousand one hundred individuals were invited to participate in the study and a total of 4,195 out of 6,739 eligible individuals completed the interview (response rate = 62.3%). The sample had a mean age of 43.2 years (SD = 13.5) and comprised 48.8% males. The characteristics of the study sample can be found in Supplementary Table 1.

### 3.1 Factor analyses

EFA indicated a four- or five-factor model as potential solutions. The five-factor model accounted for 71.7% of variance. Table 1 lists the 12 causal beliefs items. Items CS1 and CS2 loaded on the first factor. CS3, CS4, CS5 and CS6 loaded on the second factor. CS10 and CS11 loaded on the third factor. CS7 and CS8 loaded on the fourth factor. CS9 and CS12 loaded on the fifth factor. Factor loadings for all items were above 0.3. Visual scree plot was generated and factors above the point of inflection were retained (i.e., 4 factors) with eigenvalues>1. However, as the eigenvalue was less than 1 for the fifth factor, CS9 and CS12 were removed. EFA was rerun with the remaining ten items and the final four-factor model matched the original scale, namely – physical, psychosocial, personality and biogenetic causes (11) (Table 1). CFA was performed on the remaining half of the sample to confirm the factor structure. The four-factor model accounted for 64.2% of variance and provided acceptable fit to the data (RMSEA = 0.031, TLI = 0.933, CFI = 0.957, SRMR = 0.031, and χ^2^_(df)_ = 1410.12_(45)_). Overall internal reliability was 0.74.

**Table 1:** Exploratory factor analysis (EFA) factor loadings of causal beliefs scale.

Item	Factor 1 physical	Factor 2 psychosocial	Factor 3 personality	Factor 4 biogenetic
CS1: Could a virus or other infection, be a reason for these sorts of problems?	**0.74**	0.04	0.13	0.14
CS2: Could an allergy or reaction be a cause for these sorts of problems?	**0.66**	0.01	0.15	0.14
CS3: Could everyday problems such as stress, family arguments, difficulties at work or financial difficulties be a cause (for these sorts of problems)?	−0.02	**0.57**	0.19	0.05
CS4: Could the recent death of a close friend or relative be a reason for these sorts of problems?	0.02	**0.77**	0.15	0.14
CS5: Could some recent traumatic event such as a severe traffic accident be a cause for these sorts of problems?	0.08	**0.64**	0.12	0.22
CS6: Could childhood problems such as being badly treated or abused, losing one or both parents when young or coming from a broken home be a reason (for these sorts of problems)?	0.02	**0.50**	0.23	0.15
CS7: How likely is it that these sorts of problems are inherited or genetic or run in the family?	0.10	0.09	0.13	**0.42**
CS8: How likely is it that these sorts of problems are caused by a chemical imbalance in the brain?	0.12	0.15	0.13	**0.65**
CS9: How likely is it that these sorts of problems are caused by spirit possession, supernatural causes or black magic?	Removed			
CS10: Is being a nervous person likely to be a reason (for these sorts of problems)?	0.17	0.18	**0.66**	0.26
CS11: Could having a weak character be a cause (for these sorts of problems)?	0.13	0.14	**0.73**	0.11
CS12: How likely is it that these sort of problems are a punishment, retribution or karma for some previous wrongdoing or bad deeds by the person/his family?	Removed			

Values > 0.3 are highlighted in bold.

### 3.2 Descriptive statistics of the causal beliefs scale

Total scores for physical, personality, and biogenetic causes ranged from 0 to 2, while psychosocial causes ranged from 0 to 4, with higher scores indicating stronger endorsement of the type of causal belief. Across the seven mental illness vignettes, the weighted mean (SD) of causal belief factors were 0.28 (0.60), 3.11 (1.21), 0.89 (0.85), and 0.93 (0.81) for physical, psychosocial, personality, and biogenetic causes. Taking endorsement of belief as indicated by a total score≥1, psychosocial causes were most endorsed by respondents (94.4%), followed by biogenetic causes (62.6%), personality causes (57.2%) and physical causes (20.6%) (Table 2).

**Table 2:** Weighted proportion of endorsed causal beliefs.

	Total score									
	0		1		2		3		4	
	*n*	%	*n*	%	*n*	%	*n*	%	*n*	%
Physical causes										
Depression	429	70.77	96	16.56	75	12.56	–	–	–	–
Schizophrenia	481	80.87	78	13.53	42	5.54	–	–	–	–
OCD	369	61.06	122	20.46	108	17.94	–	–	–	–
Alcohol abuse	530	88.82	48	7.09	19	3.98	–	–	–	–
Dementia	489	81.34	82	13.80	27	4.80	–	–	–	–
Depression with suicidality	487	81.64	62	11.89	47	5.97	–	–	–	–
Gambling disorder	533	90.19	42	5.68	20	4.13	–	–	–	–
Psychosocial causes										
Depression	10	0.88	17	2.46	42	7.42	117	21.11	415	67.73
Schizophrenia	11	1.69	20	3.39	55	8.04	101	15.62	411	71.03
OCD	75	10.18	95	15.90	99	14.68	105	17.36	223	41.70
Alcohol abuse	20	3.86	33	3.62	70	11.21	149	23.59	325	57.60
Dementia	54	10.34	65	11.65	73	13.50	142	22.71	262	41.24
Depression with suicidality	13	1.06	22	4.28	42	6.87	109	17.94	411	69.80
Gambling disorder	47	9.47	93	14.08	124	23.22	133	20.04	195	32.60
Personality causes										
Depression	243	38.15	131	21.96	227	39.84	–	–	–	–
Schizophrenia	262	39.60	147	27.11	192	32.83	–	–	–	–
OCD	230	39.85	187	33.55	182	26.52	–	–	–	–
Alcohol abuse	279	44.06	159	23.98	160	31.89	–	–	–	–
Dementia	322	53.15	119	22.34	156	24.40	–	–	–	–
Depression with suicidality	210	37.34	138	17.54	248	44.16	–	–	–	–
Gambling disorder	267	45.42	206	35.31	120	18.69	–	–	–	–
Biogenetic causes										
Depression	218	36.24	204	30.52	175	32.97	–	–	–	–
Schizophrenia	172	27.13	233	37.47	188	33.21	–	–	–	–
OCD	185	33.53	217	35.29	187	29.10	–	–	–	–
Alcohol abuse	278	47.50	197	29.14	115	22.03	–	–	–	–
Dementia	138	22.90	209	32.44	245	43.57	–	–	–	–
Depression with suicidality	224	35.57	208	34.21	158	28.57	–	–	–	–
Gambling disorder	282	48.32	226	38.88	76	10.50	–	–	–	–

*n* and % are weighted. OCD, Obsessive-compulsive disorder.

### 3.3 Regression analyses

Table 3 shows the results of the multivariable linear regression analyses. Significant associations were found between the causal beliefs with age, gender, marital status, highest education level, monthly income, vignette type, recognition of condition, and experiences with mental illness.

**Table 3:** Multivariable linear regression for factors of causal beliefs.

	Physical (*n* = 4,107)				Psychosocial (*n* = 4,099)				Personality (*n* = 4,105)				Biogenetic (*n* = 4,057)			
	*β*	95% CI		*p*	*β*	95% CI		*p*	*β*	95% CI		*p*	*β*	95% CI		*p*
Age																
18–34 (ref)																
35–49	0.01	−0.06	0.08	0.799	−0.21	−0.34	−0.07	**0.003**	0.11	0.00	0.22	**0.044**	0.00	−0.10	0.09	0.954
50–67	0.06	−0.02	0.14	0.124	−0.26	−0.41	−0.11	**0.001**	0.17	0.06	0.29	**0.004**	−0.01	−0.11	0.10	0.863
Gender																
Female (ref)																
Male	−0.02	−0.07	0.03	0.439	−0.15	−0.25	−0.04	**0.005**	−0.01	−0.09	0.07	0.813	−0.10	−0.17	−0.03	**0.006**
Ethnicity																
Chinese (ref)																
Malay	0.02	−0.03	0.07	0.438	0.05	−0.05	0.15	0.304	−0.02	−0.09	0.05	0.618	0.01	−0.06	0.08	0.832
Indian	−0.01	−0.06	0.03	0.529	−0.08	−0.17	0.01	0.097	−0.06	−0.13	0.01	0.097	−0.02	−0.08	0.05	0.587
Others	−0.05	−0.11	0.02	0.171	0.00	−0.13	0.13	0.991	−0.07	−0.17	0.03	0.159	0.04	−0.05	0.14	0.368
Marital status																
Currently Married (ref)																
Never Married	−0.04	−0.10	0.03	0.264	0.07	−0.05	0.20	0.256	−0.12	−0.23	−0.02	**0.017**	0.01	−0.08	0.10	0.764
Separated/Widowed/Divorced	0.04	−0.09	0.17	0.518	−0.03	−0.26	0.21	0.822	−0.01	−0.16	0.15	0.919	−0.02	−0.17	0.13	0.811
Highest education level																
University (ref)																
Primary and below	0.26	0.10	0.41	**0.001**	−0.04	−0.31	0.22	0.763	0.01	−0.18	0.20	0.893	0.01	−0.18	0.20	0.912
Secondary	−0.01	−0.10	0.07	0.778	0.02	−0.14	0.19	0.790	0.01	−0.11	0.13	0.893	−0.16	−0.27	−0.04	**0.007**
Pre-university	−0.03	−0.09	0.04	0.389	0.03	−0.10	0.16	0.691	0.01	−0.09	0.11	0.827	−0.15	−0.24	−0.06	**0.002**
Employment status																
Currently working (ref)																
Unemployed	−0.05	−0.18	0.07	0.407	−0.08	−0.37	0.21	0.592	0.07	−0.13	0.27	0.509	0.19	0.00	0.38	0.050
Economically inactive	−0.07	−0.17	0.03	0.181	−0.02	−0.19	0.16	0.844	−0.13	−0.26	0.00	0.057	0.03	−0.09	0.16	0.593
Monthly income (SGD)																
$10,000 and above (ref)																
Below $2000	0.13	0.01	0.26	**0.037**	−0.22	−0.47	0.03	0.079	0.06	−0.12	0.25	0.521	−0.16	−0.33	0.02	0.077
$2000–3,999	0.10	−0.01	0.20	0.086	−0.14	−0.37	0.09	0.234	0.02	−0.14	0.19	0.779	−0.10	−0.25	0.06	0.215
$4000–5,999	0.04	−0.06	0.15	0.405	−0.10	−0.32	0.13	0.396	0.00	−0.16	0.17	0.982	−0.07	−0.22	0.09	0.403
$6000–9,999	0.05	−0.05	0.16	0.339	−0.02	−0.25	0.22	0.880	−0.04	−0.21	0.13	0.614	−0.07	−0.23	0.08	0.346
Vignette																
Schizophrenia (ref)																
Depression	0.18	0.07	0.29	**0.002**	−0.02	−0.19	0.15	0.811	0.14	−0.01	0.30	0.065	−0.20	−0.34	−0.06	**0.006**
OCD	0.36	0.24	0.47	**0.000**	−0.91	−1.12	−0.70	**0.000**	0.01	−0.13	0.15	0.849	−0.15	−0.28	−0.01	**0.033**
Alcohol abuse	−0.05	−0.14	0.04	0.313	−0.24	−0.41	−0.06	**0.009**	0.03	−0.12	0.19	0.657	−0.36	−0.50	−0.23	**0.000**
Dementia	0.04	−0.05	0.14	0.382	−0.81	−1.02	−0.59	**0.000**	−0.12	−0.27	0.03	0.110	0.05	−0.09	0.19	0.490
Depression with suicidality	0.02	−0.08	0.11	0.748	−0.05	−0.23	0.13	0.577	0.24	0.09	0.40	**0.002**	−0.22	−0.36	−0.08	**0.002**
Gambling disorder	−0.05	−0.15	0.05	0.319	−1.01	−1.22	−0.80	**0.000**	−0.10	−0.24	0.05	0.194	−0.50	−0.63	−0.37	**0.000**
Recognition of mental illness																
Did not recognize (ref)																
Mislabeled/unspecified mental illness	0.00	−0.11	0.10	0.931	0.27	0.11	0.43	**0.001**	0.04	−0.11	0.18	0.598	−0.05	−0.19	0.09	0.489
Correct recognition	−0.09	−0.16	−0.02	**0.016**	0.18	0.05	0.30	**0.008**	−0.13	−0.23	−0.04	**0.006**	0.10	0.01	0.19	**0.030**
Have you ever had problems similar to XX?																
No (ref)																
Yes	0.09	0.00	0.18	**0.048**	0.13	−0.01	0.27	0.061	0.03	−0.08	0.15	0.570	0.10	−0.01	0.21	0.083
Have you ever had a job that involved providing treatment or mental health services to a person with a problem like XX?																
No (ref)																
Yes	0.03	−0.09	0.15	0.612	−0.23	−0.45	0.00	**0.048**	−0.05	−0.21	0.11	0.528	0.17	0.04	0.30	**0.011**
Has anyone in your family or close circle of friends ever had problems similar to XX?																
No (ref)																
Yes	0.01	−0.06	0.07	0.846	−0.08	−0.21	0.05	0.227	0.06	−0.03	0.15	0.196	0.18	0.10	0.26	**0.000**

Unadjusted *R*^2^ values ranged from 0.059–0.153. OCD, Obsessive-compulsive disorder. Ref, reference group. XX, vignette character. *p* < 0.05 are highlighted in bold.

#### 3.3.1 Sociodemographic differences

Respondents with primary education and below (*β* = 0.26) and those with monthly income below $2000 (*β* = 0.13) were more likely to endorse physical causes as compared to those with university education and those with monthly income of $10,000 and above. Older respondents were less likely to endorse psychosocial causes (35–49: *β* = −0.21; 50–67: *β* = −0.26) but were more likely to endorse personality causes (35–49: *β* = 0.11; 50–67: *β* = 0.17) as compared to those aged 18–34 years. Respondents who were never married (*β* = −0.12) were less likely to endorse personality causes as compared to those currently married. Males were less likely to endorse psychosocial (*β* = −0.15) and biogenetic causes (*β* = −0.10). Those with secondary education (*β* = −0.16) and pre-university education (*β* = −0.15) were less likely to endorse biogenetic causes as compared to those with university education.

#### 3.3.2 Vignette type

Compared to those who received the schizophrenia vignette, respondents who received the depression (*β* = 0.18) or OCD (*β* = 0.36) vignette were more likely to endorse physical causes. Respondents who received the OCD (*β* = −0.91), alcohol abuse (*β* = −0.24), dementia (*β* = −0.81), or gambling disorder (*β* = −1.01) vignette were less likely to endorse psychosocial causes. Only respondents who received the depression with suicidality (*β* = 0.24) vignette were more likely to endorse personality causes. Respondents who received the depression (*β* = −0.20), OCD (*β* = −0.15), alcohol abuse (*β* = −0.36), depression with suicidality (*β* = −0.22), or gambling disorder (*β* = −0.50) vignette were less likely to endorse biogenetic causes as compared to those who received the schizophrenia vignette.

#### 3.3.3 Recognition of condition

Compared to those who did not recognize the condition in the vignette, respondents with correct recognition were less likely to endorse physical causes (*β* = −0.09) and personality causes (*β* = −0.13), but more likely to endorse psychosocial causes (*β* = 0.18) and biogenetic causes (*β* = 0.10). Those who mislabeled/did not specify the condition (*β* = 0.27) were more likely to endorse psychosocial causes.

#### 3.3.4 Experience with mental illness

Respondents who had problems similar to the vignette character were more likely to endorse physical causes (*β* = 0.09). Respondents who had a job that involved providing treatment or mental health services to a person similar to the vignette character were less likely to endorse psychosocial causes (*β* = −0.23) and more likely to endorse biogenetic causes (*β* = 0.17). Those with family members or close friends with problems similar to the vignette character were more likely to endorse biogenetic causes (*β* = 0.18).

## 4 Discussion

This study examined the causal beliefs of mental illness among the Singapore general population. A four-factor structure of the causal beliefs scale was derived and validated and several key predictors were found.

Unlike the first study in 2015, factor analyses in the current study revealed biogenetic causes as a causal belief factor. The change in factor structure could be due to the increase in the number of items relating to biogenetic causes whereby two items were used instead. However, the fifth factor relating to supernatural causes did not meet the criteria despite the addition of a second item. While supernatural causes are not part of the original causal beliefs scale, an item was first included in the 2015 study due to its cultural relevance in Asian populations. For instance, supernatural causes were endorsed by 53% of psychiatric patients in Malaysia (34), while a number of participants believed that mental illness is caused by God’s punishment for past sin (21.3%) and witchcraft (27.2%) even though they were from urban areas in India (35). Despite so, supernatural causes were not established as a factor in the 2015 study to which the authors attributed it to the use of a single item to represent supernatural beliefs (23). Supernatural beliefs remain very much culturally relevant in the present context, considering that traditional treatment (e.g., traditional Chinese medicine practitioner, Ayurvedic- or Jamu-based treatment) and religious advisors (e.g., priest, pastor, or Ustadz) are perceived as helpful interventions for individuals with a mental illness in Singapore (36). As such, another item on supernatural causes was added in the current study with the aim to strengthen its reliability and validity as a factor. A plausible reason why increasing the number of items on supernatural causes did not improve its factor loading in the current study could be that the new item relating to ‘punishment, retribution or karma’ was more relevant to concepts of divine beings and religious beliefs, while the item ‘spirit possession, supernatural causes or black magic’ relates more to paranormal origins (12). A qualitative study in Indonesia explored alternative causal beliefs and outlined three different but related themes involving demonic possession and supernatural forces, sins committed by self or family members, and witchcraft or black magic (37). Apart from the possibility that the items do not represent supernatural causes as a construct adequately, the different ethnicities and religions in Singapore might have also contributed to nuance differences in the interpretation of these statements, and thus the factor could not be established. Given that help-seeking behaviors depend on the type of causal belief (2, 12, 38), it would be beneficial for future studies to include more items related to supernatural causes or consider these two types of beliefs separately.

People’s perceptions of mental illness etiology depended on the type of condition depicted in the vignette that was randomly allocated to them. Schizophrenia was used as the comparison group in the analyses due to considerable evidence on its causal beliefs that acknowledged the condition’s genetic predisposition and psychosocial risk factors (9, 11, 18). Indeed, compared to schizophrenia, biogenetic causes were less likely to be endorsed for all other mental illnesses except dementia, and psychosocial causes were less likely to be endorsed for all other mental illnesses except depression and depression with suicidality. Dementia is a common neurodegenerative condition afflicting one in 11 older adults in Singapore (26). The public’s understanding of dementia has also greatly improved consequent to many public education initiatives, and hence there is a strong awareness of the underlying biological causes of dementia among the general population. As for depression, respondents showed an overwhelming inclination for psychosocial causes with endorsement rates as high as 98.7% and 98.9% for depression and depression with suicidality respectively (where total scores ≥ 1). This trend is reflected in the literature as well, where social and psychological factors such as normal up and downs of life, upbringing, stressful life events, and cognition were more commonly cited as causes of depression (13, 39).

Interestingly, physical factors were positively associated with depression and OCD. A plausible explanation for perceiving a virus or an allergy reaction as causes for OCD could be due to the recent COVID-19 pandemic which normalized frequent handwashing and sanitization of environment (40). Thus, respondents might have viewed the OCD symptoms and anxiety over contamination depicted in the vignette to be caused by physical factors like viruses. On the other hand, the association between physical factors and depression could be due to the presentation of somatic symptoms such as fatigue, lack of appetite and sleep problems in the vignette. It is not uncommon for people to misinterpret such somatic symptoms of depression to stem from physical causes and therefore to first seek medical help (4, 41). In addition, while no associations were found between personality beliefs and most mental illnesses, the positive association with depression with suicidality is concerning. It suggests that suicidality is perceived to be caused by character and personality flaws, which signals the presence of stigma and could prevent individuals with suicidality from seeking help in fear of being perceived as weak (5, 6). Taken altogether, these findings showed that the public is aware of the role of biogenetic and psychosocial factors in the etiology of mental illness, however, certain aspects still require attention and should be prioritized.

In line with the literature, recognition was linked to better understanding of the etiology of mental illness. These respondents endorsed more of biogenetic and psychosocial beliefs and less of physical and personality beliefs (2). Experience with mental illness was a significant predictor of causal beliefs too, especially for biogenetic beliefs. Respondents who had a mental health-related profession were more likely to endorse biogenetic causes and less of psychosocial causes (15, 19, 20), while those with family members or close friends with mental illness were more likely to endorse biogenetic causes (18). The latter finding differed from studies that only looked at schizophrenia (19), which is likely due to the analysis being based on the consolidated responses across all mental illness vignettes. However, unlike other studies, it is less clear why respondents who had ever experienced similar problems were more likely to endorse physical causes. A potential reason, according to anecdotal accounts, is that patients may misinterpret psychiatrists’ explanations of what mental illness is. Explanations such as mental illness is a “brain disorder” or is a result of “imbalances in brain chemistry” could have led patients to view mental illness as something similar to physical illness, and thus respondents who identified as having experienced similar problems could also have attributed the causes of mental illness to physical factors instead. On the other hand, self-identification as having a mental illness is linked to better mental health knowledge and help-seeking intentions, but somatization tendencies and stigma can impede recognition and help-seeking behaviors among those currently untreated (3, 42). Somatization tendencies refer to when individuals tend to experience and present their psychopathology or underlying psychological distress through somatic concerns like headaches, nausea, and chest pains which are not linked to any physical health conditions. Such situations are common among those with mood and anxiety-related disorders and are also observed in Asian populations more often than non-Western cultures due to reasons not limiting to lack of mental health awareness, cultural norms, fear of being labeled as “mentally ill” or being ostracized (41, 43, 44). It is therefore possible that respondents who reported having similar problems as the vignette character were unwilling to acknowledge their symptoms as psychiatric-related or needed a socially acceptable reason to seek medical help (41, 43), though a qualitative inquiry is needed to verify this claim. Regardless, this finding also implied that people struggling with mental health problems may first approach general practitioners or family physicians for their condition and as such, support should be given to primary care providers to help them detect and refer patients that require psychiatric help.

Several sociodemographic variables were also significantly associated with causal beliefs. Older age was associated with higher endorsement of personality causes and lower endorsement of psychosocial causes. This finding could be a result of lower mental health literacy which is commonly observed among older adults (4, 14). Older individuals could also be less open to new information due to preconceived notions that are perpetuated by stigma toward persons with mental illness (45). Additionally, although studies suggest that men were more likely to endorse personality causes than women (18), this was not seen in the present study. Instead, male respondents were found to be less likely to endorse psychosocial (4) and biogenetic causes (11). Lower education qualifications and income are often associated with less mental health knowledge too (4), which would explain why these respondents viewed mental illness as caused by physical factors. On the contrary, those with university education were more likely to endorse biogenetic causes, possibly due to the emphasis on scientific explanations in tertiary institutions that is in accordance with the medical field perspectives.

The strengths of this study comprise the inclusion of a large and representative sample of the general population that was derived from a national administrative database and the use of robust methodology that was replicated from the previous study which enables comparison with earlier findings and other studies with similar methodological approach. The use of the split-sample EFA/CFA validation analytical method also allow the sample to be randomly split into two groups before performing factor analysis and thus reducing bias associated with over-fitting and ensuring better generalizability of the results to the general population. However, some limitations should be considered such as the cross-sectional nature of the study whereby causality cannot be determined. Also, as this paper focused on the factor structure and predictors of causal beliefs, it is therefore unclear whether endorsing specific beliefs lead to better social outcomes such as lowered stigmatizing attitudes and increased help-seeking behaviors. Lastly, causal beliefs are multifactorial in nature, and individuals can endorse more than one type of explanation for each mental illness (13, 14, 20). As such, future studies may consider studying profiles of causal beliefs to better understand its link to outcomes of social inclusion and stigma.

### 4.1 Implications

The etiology of mental illness is often addressed in many MHL campaigns in Singapore. Because causal beliefs are intricately related to stigma and help-seeking (5, 6, 38), impacting the public’s views on the causes of mental illness can influence their attitudes toward persons with mental illness and toward seeking appropriate treatment. Phrasing of campaign messages, therefore, have a significant ripple effect and to achieve the desired results, the existing MHL landscape must be taken into account. In this study, the majority of the Singapore general population were found to endorse psychosocial causes of mental illness, with about two-thirds also endorsing biogenetic and personality causes.

While it is beyond the scope of the current paper to establish direct links between causal beliefs, stigma and help-seeking, the literature has offered some insights. Information on the causes of mental illness given to the public should continue to be scientifically accurate but biogenetic-related explanations should not be overstated (46). This is because extensive research on biogenetic beliefs in recent years have revealed mixed effects on stigma. The medicalization of mental illness put forward the notion that mental illness can be explained by biological and genetic factors and medical interventions are possible. As such, the introduction of biogenetic concepts was particularly effective in improving people’s understanding and attitudes in the nineteenth century where information on mental illness was limited and treatment centered around exorcism, imprisonment in mental asylums and execution (47). This perspective reduces blame towards the individual and increases uptake of professional help (10, 47, 48), particularly among Asian countries (48). Yet at the same time, biological explanations promote genetic essentialism and prognostic pessimism whereby mental illness is viewed as permanent and determined from birth, and that treatability and recovery is unlikely (46, 48). Such beliefs also create the impression that persons with mental illness inherently lack individual control and thus they are often perceived as dangerous and unpredictable. As a result, they may be avoided and become socially distanced from others (5, 6, 46, 47). Furthermore, biogenetic explanations can have varying influence across different mental illness, with studies reporting people having less tolerant attitudes toward individuals with depression and schizophrenia as compared to those with alcohol dependence (46, 49). To minimize unintended consequences, campaign messages should instead emphasize the possibilities of recovery and treatability of mental illness alongside biogenetic explanations and adjust the level of emphasis according to the specific condition (47, 50). Conversely, although psychosocial beliefs are associated with lower stigma (38, 46), informal sources of help (e.g., family, friends) become the first choice to seek help from (2, 39). The drawbacks of informal help include dismissive reactions that trivialize the person’s struggles or provision of inaccurate information. However, these effects may be negated by improving MHL levels among the general public as these informal sources can become a gateway to professional help through appropriate referral (2, 38). Reducing the relatively high endorsement rates of personality beliefs should also be a key target for campaigns in Singapore. Holding personality beliefs is strongly associated with stigma perceptions of ‘weak-not-sick’ and dangerousness (5) and such views in turn diminish help-seeking intentions (3). Campaign messages highlighting how mental illness is common and focusing on seeing the person beyond the diagnosis can be further complemented with contact-based interventions which would better increase people’s understanding of persons with mental illness and engender more positive attitudes toward mental illness (17, 50, 51).

## 5 Conclusion

The general population in Singapore perceive mental illness to be largely caused by psychosocial reasons. Biogenetic and personality factors were also frequently recognized as possible causes. As campaigns continue to address gaps in MHL levels, the relatively high endorsement of biogenetic and personality causes raises some concerns. Given their strong associations with stigma and help-seeking, careful promotion on the etiology of mental illness is warranted to avoid conveying unintended messages. Relevant agencies and organizations can build on the findings of this study on the public’s causal beliefs to better curate their campaigns and outreach initiatives.

## Data Availability

The data supporting the findings of this study are available from the corresponding author, YT, upon reasonable request.
