# Supplementary material for: Factor structure and predictors of causal beliefs about seven mental illnesses among the Singapore general population
Source: Front Public Health. 2025 Jul 9;13:1612820. doi: 10.3389/fpubh.2025.1612820 (PMC12283601; doi:10.3389/fpubh.2025.1612820)
Supplement: Supplementary file 1 [file Table_1.docx]

Supplementary Table 1. Characteristics of study sample

|  | **N** | **Weighted %** |
| --- | --- | --- |
| **Age groups** |  |  |
| 18-34 | 1377 | 31.7 |
| 35-49 | 1315 | 31.6 |
| 50-67 | 1503 | 36.7 |
| **Gender** |  |  |
| Female | 2149 | 51.2 |
| Male | 2046 | 48.8 |
| **Ethnicity** |  |  |
| Chinese | 1150 | 73.8 |
| Malay | 1347 | 13.3 |
| Indian | 1322 | 9.2 |
| Others | 376 | 3.6 |
| **Marital status** |  |  |
| Never Married | 1278 | 32.9 |
| Currently married | 2630 | 60.3 |
| Separated/Divorced/Widowed | 287 | 6.8 |
| **Highest education** |  |  |
| Primary and below | 318 | 7.3 |
| Secondary | 976 | 21.6 |
| Pre-university | 1454 | 30.9 |
| University | 1447 | 40.3 |
| **Employment status** |  |  |
| Currently employed | 3322 | 80.9 |
| Unemployed | 179 | 4.0 |
| Economically inactive | 694 | 15.0 |
| **Monthly personal income** |  |  |
| Below 10,000 | 1433 | 30.2 |
| 2,000 to 3,999 | 1202 | 26.0 |
| 4,000 to 5,999 | 724 | 19.0 |
| 6,000 to 9,999 | 482 | 15.1 |
| Above 10,000 | 294 | 8.1 |
| **Recognition of vignettes** |  |  |
| Correct recognition | 2592 | 63.3 |
| Mislabelled/unspecified mental health condition | 413 | 9.3 |
| Did not recognize | 1190 | 27.4 |
| **Vignettes** |  |  |
| Depression | 602 | 14.4 |
| Schizophrenia | 602 | 14.5 |
| OCD | 600 | 14.2 |
| Alcohol abuse | 599 | 14.3 |
| Dementia | 599 | 14.2 |
| Depression with Suicidality | 598 | 14.2 |
| Gambling disorder | 595 | 14.3 |
| **Have you ever had problems similar to XX?** | | |
| Yes | 608 | 14.5 |
| No | 3583 | 85.4 |
| **Have you ever had a job that involved providing treatment or mental health services to a personal with a problem like XX?** | | |
| Yes | 305 | 6.0 |
| No | 3882 | 93.6 |
| **Has anyone in your family or close circle of friends ever had problems similar to XX?** | | |
| Yes | 1036 | 25.2 |
| No | 3150 | 74.7 |
| Note. ^#^Pre-university include A Levels, Polytechnic Diploma and ITE Certificate. | | |
